# Recent Advances in Understanding σ70-Dependent Transcription Initiation Mechanisms

**DOI:** 10.1016/j.jmb.2019.04.046

**Published:** 2019-09-20

**Authors:** Abhishek Mazumder, Achillefs N. Kapanidis

**Affiliations:** Biological Physics Research Group, Clarendon Laboratory, Department of Physics, University of Oxford, Oxford, UK

## Abstract

Prokaryotic transcription is one of the most studied biological systems, with relevance to many fields including the development and use of antibiotics, the construction of synthetic gene networks, and the development of many cutting-edge methodologies. Here, we discuss recent structural, biochemical, and single-molecule biophysical studies targeting the mechanisms of transcription initiation in bacteria, including the formation of the open complex, the reaction of initial transcription, and the promoter escape step that leads to elongation. We specifically focus on the mechanisms employed by the RNA polymerase holoenzyme with the housekeeping sigma factor σ^70^. The recent progress provides answers to long-held questions, identifies intriguing new behaviours, and opens up fresh questions for the field of transcription.

## Graphical Abstract

**Figure:**
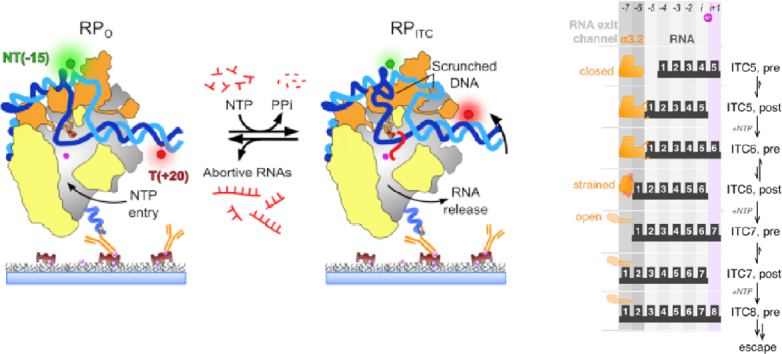

## Background

Transcription is a fundamental process in all living organisms and serves as the first step in the flow of information from genes to functional molecules such as proteins or RNAs. Transcription in all organisms is highly regulated to ensure the right genes are expressed at the right places and in the amounts required for proper functioning of the cell [1].

The protein machine at the heart of transcription is the RNA polymerase (RNAP), which can function alone or with other co-factors to copy information encoded in DNA to synthesise a RNA molecule. Despite the fact that the RNAP can, in principle, perform transcription from any DNA sequence, transcription was shown to initiate from specific DNA sequence elements called promoters inside the bacterial cell [2], [3], [4], [5]. This specific initiation requires a protein cofactor named sigma (σ) factor; the σ factor is a key component that associates with the RNA polymerase core enzyme to yield a RNA polymerase holoenzyme, which is the form of the enzyme required for specific transcription initiation [6]. A separate study also found that the σ factor was only involved during transcription initiation, and it dissociated from RNAP after this stage to become available to bind another molecule of core RNAP [7]. Since then, it has been established that all bacteria have a “housekeeping” σ factor, which is involved in majority of transcription initiation events in the cell. The two most well studied of the housekeeping sigma factors are the σ^70^ (so named due to its molecular weight of 70 KDa) in *E.coli* and the σA in *T. thermophilus*. In the early 1980s, Losick and coworkers discovered that most bacteria contain multiple proteins having high sequence homology to σ factors, and these different types of protein cofactors may associate with the RNA polymerase and result in switching between sets of promoters thus altering the global transcriptional landscape of the cell [8]. The sigma-like factors discovered in this work has since been classified as alternative sigma factors and has been shown to be mostly concerned with coping of different types of stresses experienced by bacteria. Here, we review the recent progress on the σ^70^/σ^A^ dependent transcription initiation regulation and mechanism arising from biochemical, structural and single molecule studies.

## Bacterial RNAP, promoter architecture and structural organisation of the open complex

### Bacterial RNAP core enzyme

RNAP is conserved among all organisms. The bacterial, archaeal and eukaryotic RNAP are all members of a conserved protein family, named the “multi-subunit RNAP family” [9], [10]. The bacterial RNA polymerase core enzyme is a multi-subunit molecular machine having five subunits (αI, αII, β, β’ and ω) and contains all determinants needed for non-specific transcription initiation and elongation.

**Fig. 1:**
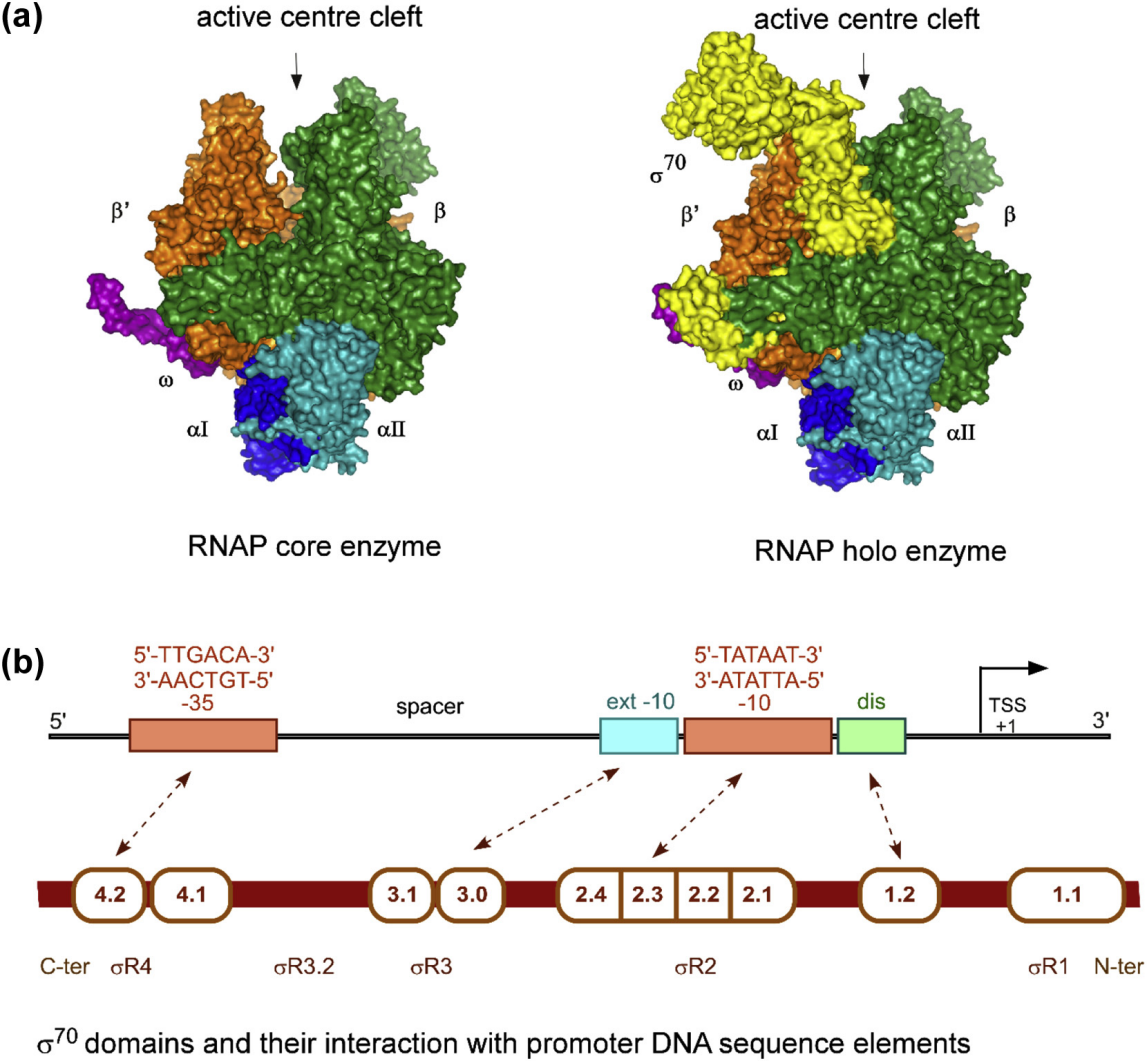
A) Structure of bacterial RNAP core enzyme (*left*) and holoenzyme (*right*); B) Schematic of a bacterial promoter showing consensus − 10 and − 35 elements, extended − 10 and discriminator elements and their interaction with different modules of σ^70^.

The first structure of a complete multi-subunit RNAP core enzyme from *T.Aquaticus* was solved in the late 1990s, followed by the structure of the RNAP core enzyme from *E.coli* [10], [11]. Significant advances in structural biology in the following two decades led to the determination of several high resolution structural studies of the bacterial RNA polymerase sigma factors, core enzymes, holoenzymes, RNAP-promoter complexes, and transcription initiation complexes; these structures have substantially enriched our structural understanding of this fascinating molecular machine [11], [12], [13], [14], [15], [16], [17], [18], [19], [20]. Overall, the structures revealed a RNAP core enzyme having dimensions of ∼ 150 Å X ∼ 100 Å X ∼ 100 Å, that adopt a crab-claw shape, with two “pincers” of the “claw” defining the active-centre cleft (Fig. 1A) and which has an active-centre catalytic Mg^2+^ at its base. The β’ subunit makes up one pincer, termed the “clamp,” and part of the base of the active-centre cleft. The β subunit makes up the other pincer and the other part of the base of the active-centre cleft. In addition to the active-centre cleft, the RNA polymerase structure has two more channels: the secondary channel (which allows access to the incoming NTPs), and the RNA exit channel (which can accommodate a growing nascent RNA chain).

### σ^70^ and the RNAP holoenzyme

Transcription initiation from *E.coli* promoters requires RNAP association with *σ* factor; however, free σ^70^ does not bind DNA [21]. Studies on σ^70^ using crosslinking and FRET-based methods showed that free σ^70^exists in a compact conformation and upon association with core RNA polymerase, σ^70^ undergoes a large conformational change that unmask regions in the protein that could bind DNA [22], [23]. The first high resolution structures of the σ^70^ family of proteins came from X-ray crystallography studies of different domains of σ^70^ (from *E.coli*) and σ^A^ (from *T. thermophilus*) [24], [25]. Soon after a systematic-FRET study measuring multiple distances between specific subunits of σ^70^ and the RNAP core outlined the organisation of σ^70^ with respect to the core enzyme [26]. In recent years several high-resolution structures of RNAP holoenzyme have also outlined different modular domains of σ^70^, their interactions with the core enzyme, and their organisation in the holoenzyme [13], [14]. Together, these studies revealed that σ^70^ contains five conserved regions: σR1.1, σR2, σR3, σR3.2 (also known as σR3/σR4 linker), and σR4. The σR2, σR3 and σR4 are structured, modular domains linked by flexible linker elements, while σR1.1 and σR3.2 are unstructured negatively charged domains [25]. The interface between the core subunits and σ in RNAP holoenzyme is formed by three modular domains, σR2, σR3, and σR4. The σR2 interacts with β’ pincer, in and above the RNAP active-centre cleft, σR3 interacts with the base of the β flap, and σR4 interacts with the tip of the β flap. The σR1.1 is a highly negatively charged segment, which serves as a mimic of the negatively charged DNA and is located in the RNAP active-centre cleft [27], [28] in the RNAP holoenzyme but is located outside the active center cleft in the RNAP-promoter open complex. This module, only found in housekeeping σ factors, therefore needs to be displaced to permit active center cleft access to promoter DNA, and has been described as the “gatekeeper” of the RNAP active center. Hence, σR1.1 prevents RNAP from stable non-specific association with non-promoter DNA sites and is displaced from the active center cleft when sequence specific contacts are made between the polymerase and the promoter DNA. The σR3.2 is also a negatively charged flexible element and has been implicated in crucial points during initial transcription [12], [19], [29].

### Bacterial promoters

The specific DNA sequences from which the bacterial RNAP holoenzyme initiates transcription are called promoters [4], [5]. Early biochemical studies demonstrated that productive transcription rates (i.e. synthesis of full-length RNA products from a given promoter) could vary over 10,000 fold for different promoter sequences [30]. The initiation rates at a specific promoter sequence can also vary depending on external conditions (temperature, salt concentration) or the presence of other protein cofactors [31], [32].

For σ^70^-dependent transcription initiation, two main consensus promoter sequences have been identified: the − 35 hexamer (5’-TTGACA-3′) and the − 10 hexamer (5′-TATAAT-3′), where numbers represent position upstream of the transcription start site (denoted as + 1) [2]. It is important to note that almost none of the naturally occurring promoters have the consensus sequence described above. The − 35 and − 10 elements are separated by a non-sequence-specific 16–19 bp spacer region, with the consensus spacer length being 17 bp (Fig. 1B) [30], [33].

In addition to these elements, it has been shown that DNA upstream of the − 35 hexamer (− 40 to − 60; UP element) could play an important role in establishing contact with the αCTD of the core RNAP and may have significant effect on rates of open complex formation and transcription initiation [34], [35]. The entire UP element consists of two sub-sites (distal and proximal) which contact the two αCTDs and induce bending and wrapping of upstream DNA on RNAP. For some promoters, an extended − 10 element (consensus: 5′-TGTG-3′) has also been implicated in making specific contacts with σ^70^, resulting in increased open complex lifetimes [36], [37]. Parts of the downstream region between the − 10 element and the transcription start site has been designated as the discriminator region and has been shown to be important for the regulation of open complex lifetime [33], [38]. Further downstream lies the core recognition element (− 4 to + 2; CRE) which is involved in making contacts with the core RNAP; although most of the CRE bases contact RNAP, no consensus sequence has been determined [19].

### Open Complex (RP_o_)

The structural organisation of the RNAP and promoter DNA in the open complex (RP_o_) has been extensively studied using crosslinking, systematic FRET, crystallography and cryo-EM. The studies show that in the open complex, the negatively charged domain σR1.1 is displaced from the RNAP active site, which then accommodates single strands of DNA in the active site cleft [19], [39], [40], [41]. The structures also show that the structural modules σR2, σR3, and σR4 bind to promoter − 10 elements (σR2.3), extended − 10 elements (σR3.0) and − 35 (σR4.2) elements respectively (Fig. 1B). A helix-turn-helix motif of σR4.2 makes contact with the − 35 bases and for some specific promoters (such as λ P_RM_) may interact with adjacent DNA-bound transcription factors (such as the phage λ cI repressor). The σR2.3 interacts with the − 10 region of the promoter, where it makes specific contacts with bases at − 11 and − 7 by flipping them into proteins pockets in σ^70^, while it interacts non-specifically with other bases along DNA. The σR1.2 element contacts a “discriminator” element in the non-template strand of the promoter just downstream of the − 10 element (Fig. 1B) [19]. Further, it has been observed that the long flexible domain σR3-σR4 linker contains a small loop-like element protruding towards the active centre; this element has been dubbed as the σR3.2 finger and has been shown to make contacts with the template strand just upstream of the polymerase active site [19], [41].

## Mechanism of open complex formation

**Fig. 2:**
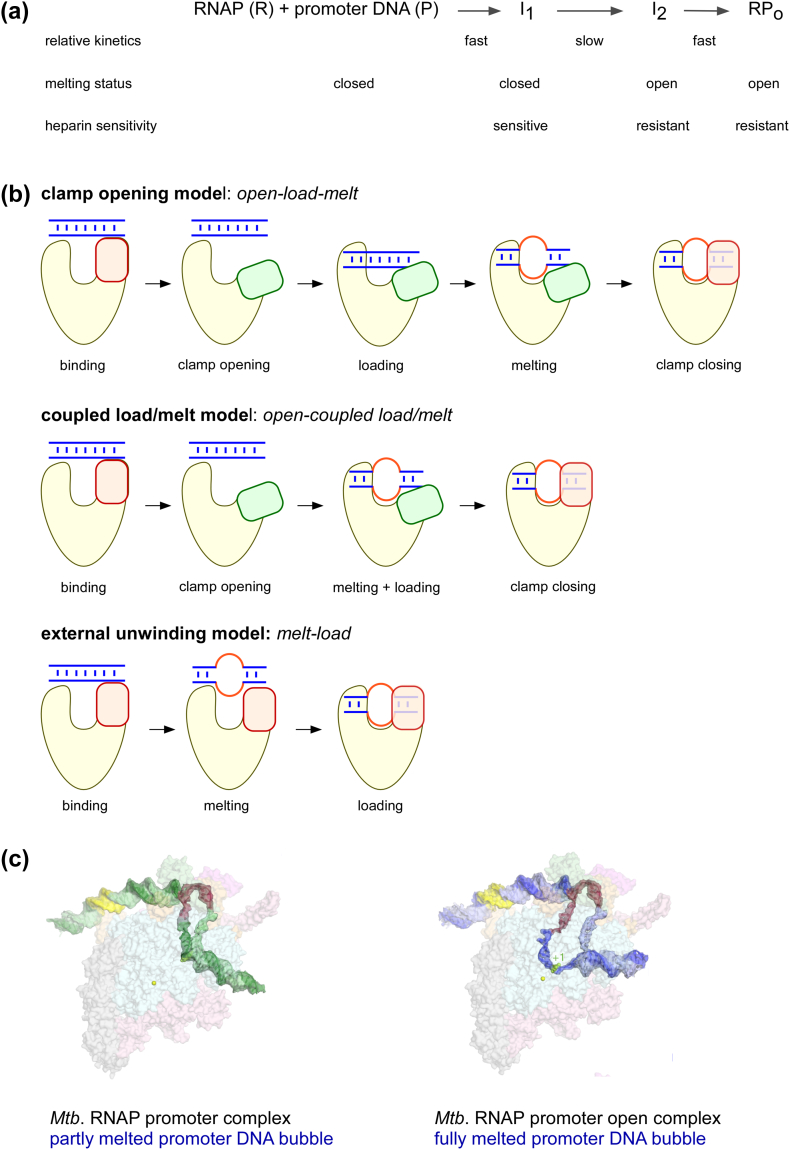
A) Schematic describing intermediates in open complex pathway showing status of promoter melting, heparin sensitivity and relative kinetic profiles of individual steps. B) schematic showing models for promoter melting and active centre cleft loading of DNA: clamp opening model (*top*), couple melt and load model (*middle*) and external unwinding model (*bottom*); in all the models the RNAP clamp is depicted in red (for closed clamp) and green (for open clamp), double stranded DNA is in blue and melted single stranded DNA is in orange; C) Structure of Mtb RNAP-promoter intermediate complex showing a partially melted DNA bubble (*left*) and an Mtb RNAP open complex with fully melted DNA bubble (*right*) (Adapted from Boyaci etal, Nature, 2019 [57]; used with permission).

The sequence of events and conformational changes leading to the formation of a transcription-competent open complex has been an intense area of research over the last three decades. Structures of promoter-RNAP complexes, single-molecule studies and real-time/low-temperature foot printing experiments have revealed the existence of several intermediates that lie on the pathway to the final open complex (RP_o_). Although the number of conformations identified and the kinetics of the individual steps at different promoters may vary, an overall picture has emerged which identifies three important steps: the initial binding of RNAP holoenzyme to promoter DNA resulting in formation of a heparin-sensitive early intermediate I_1_ where the promoter DNA is in double-stranded form (heparin is a poly anion commonly used in transcription initiation studies as a competitor of promoter DNA), a slow isomerisation of I_1_ to a heparin-resistant intermediate I_2_ with a melted open DNA bubble (− 11 to + 2), followed by transformation of the initial unstable open bubble intermediate I_2_ to the final stable open complex (RP_o_) (Fig. 2A) [31], [32], [42].

The slow rate-limiting I_1_ > I_2_ isomerisation step is only weakly dependent on salt concentration; it has thus been proposed that opening of the DNA bubble is not purely driven by the thermal dynamics of the dsDNA, but RNAP must play an active role in this step [31]. Most studies investigating this initial melting step propose that nucleation of promoter melting is initiated by RNAP following sequence-specific interaction with the highly conserved base at − 11 position of the non-template strand: -11A [43], [44]. The interaction results in flipping out the -11A base of the non-template strand into a protein pocket of σR2 where several aromatic amino acid residues F419, Y430,W433 and W434 stabilise the flipped-out conformation of the base. In particular, residue W433 uniquely positions itself to drive a wedge between the flipped-out base and the DNA [45]. This initial interaction of the − 10 element with σR2 results in a 90° bend in the DNA, thus moving the downstream DNA segment towards the RNAP active-centre cleft [45].

The final conversion of late intermediate (I_2_) to stable open complex (RP_o_), however, vary greatly with urea and salt concentrations. It has been proposed that this may indicate large scale conformational changes in the RNAP involving folding of β’ jaw and other downstream mobile elements (DME) on the downstream promoter DNA duplex, as well as motions of the RNAP clamp that trigger closure of the active center cleft leading to formation of final RNAP-promoter open complex [46], [47].

Despite the progress, the exact sequence of conformational changes resulting in the transition between the intermediates on -path to the open complex have remained puzzling. The crystal structures of RP_o_ and of RNAP initially transcribing complexes (RP_itc_) demonstrate that the polymerase accommodates single strands of DNA in the active center cleft. Access of double-stranded DNA to the active-centre cleft of RNA polymerase is restricted due to the narrow width of the cleft (< 20 Å) and the interactions of σR2 in and above the active centre cleft (Fig. 1A). This raises the intriguing question of how and when double stranded promoter DNA melts and single strands of DNA enter the RNA polymerase active site.

Two main class of models have been proposed to describe how the transcription bubble is formed in the open complex. The first is the “clamp-opening” model (also referred to as the “*open-load-melt*” model), which proposes that the RNAP active-centre cleft opens via a swinging motion of the β’ clamp, allowing double-stranded DNA to enter (Fig. 2B; top); in this model, promoter melting occurs inside the cleft, followed by closing of the cleft and formation of the final RP_o_. The “clamp-opening” model is supported by low-temperature and real-time foot printing studies that suggest that an intermediate during RPo formation contains double-stranded DNA *inside* the RNAP active-centre cleft [48], [49], [50]. The second model is the “external unwinding” model (also referred to as the “*melt-load*” model), which proposes that the melting propagates *outside* the active centre cleft, and that single-strands of unwound DNA enter the active centre cleft without any obligatory clamp opening (Fig. 2B; bottom). This model is supported by real-time kinetic experiments and simulation studies investigating promoter melting [51], [52].

A major difference between the two models for the transcription bubble formation involves the presence or not of conformational changes of the RNAP clamp. Early structural studies identified that the RNA polymerase clamp can exist in different conformations including “open” conformations that would allow for entry of ds-DNA in the active centre cleft and “closed” conformations that would permit entry of DNA only in the single stranded form [11], [13]. Recent single-molecule FRET studies of the clamp conformation in diffusing and surface-immobilised RNAP molecules revealed that the clamp exists in multiple conformational states, and can switch between an “open”, “closed” and a “partly closed” conformation [53], [54]. The same studies also showed that several RNAP inhibitors that bind to a “switch” region at the base the clamp, may lock the clamp in a particular conformation; e.g., the antibiotic lipiarmycin (Lpm) binds to the switch region and locks the clamp in an “open” conformation, while RNAP inhibitors myxopyronin (Myx) and corallopyronin (Cor) lock the clamp in a “closed” conformation [54], [55]. The facts that the clamp is mobile in solution, and that abrogation of clamp conformational dynamics inhibits RNA polymerase activity and open complex formation raise the possibility that RNAP may exploit this flexibility during RP_o_ formation. Recent structural studies of RNAP-σ54 holoenzyme promoter complexes revealed a structure of RNAP with clamp wide open and both strands of promoter DNA inside the cleft. The authors propose that this maybe a possible on-pathway intermediate to the final open complex and have put forward a “*coupled melt-load model*” based on these structures (Fig. 2B, middle) [56]. More recently cryo-EM studies on Mtb RNAP holoenzyme promoter complexes identified an “intermediate” with a partially melted promoter bubble. The authors also used, a switch region inhibitor corallopyronin (Cor) to lock the clamp in a “closed” conformation and show that it traps a similar RNAP-promoter complex in a partially melted DNA bubble conformation (Fig. 2C) [57]. It is currently unclear whether these complexes with a partially melted promoter DNA are true on-pathway intermediate, especially since there has been no evidence from single molecule or kinetic studies showing that partially melted complexes like the ones observed in this study could proceed to the open complex. In terms of the mechanism of open complex formation, this work indicates that the initial nucleation of melting may proceed without any need of clamp opening. The authors propose based on these structures that for the final melting and loading of the downstream region of the promoter DNA (− 3 to + 2), clamp needs to open and close; as a result, the prevention of clamp opening by Cor, leads to the blocking of RP_o_ formation.

A direct observation of promoter melting and clamp opening on-pathway to an open complex has not been possible yet. Single-molecule studies monitoring conformational dynamics of the promoter open bubble have observed inter-conversion between different promoter conformations, but the identity of these intermediate conformations could not be established unambiguously in these studies. Similar studies monitoring clamp conformation in these complexes have failed to observe any switching between conformational states in the RNA polymerase clamp, which stays stably closed throughout [54], [58], [59]. The exact mechanism of open complex formation therefore still remains unresolved. It is also worth noting that it is possible that a single general mechanism may not describe open complex formation at all promoters and differences in sequence of promoters may cause subtle to major differences in the mechanism of open complex formation at different promoters.

## Mechanism of initial transcription

**Fig. 3:**
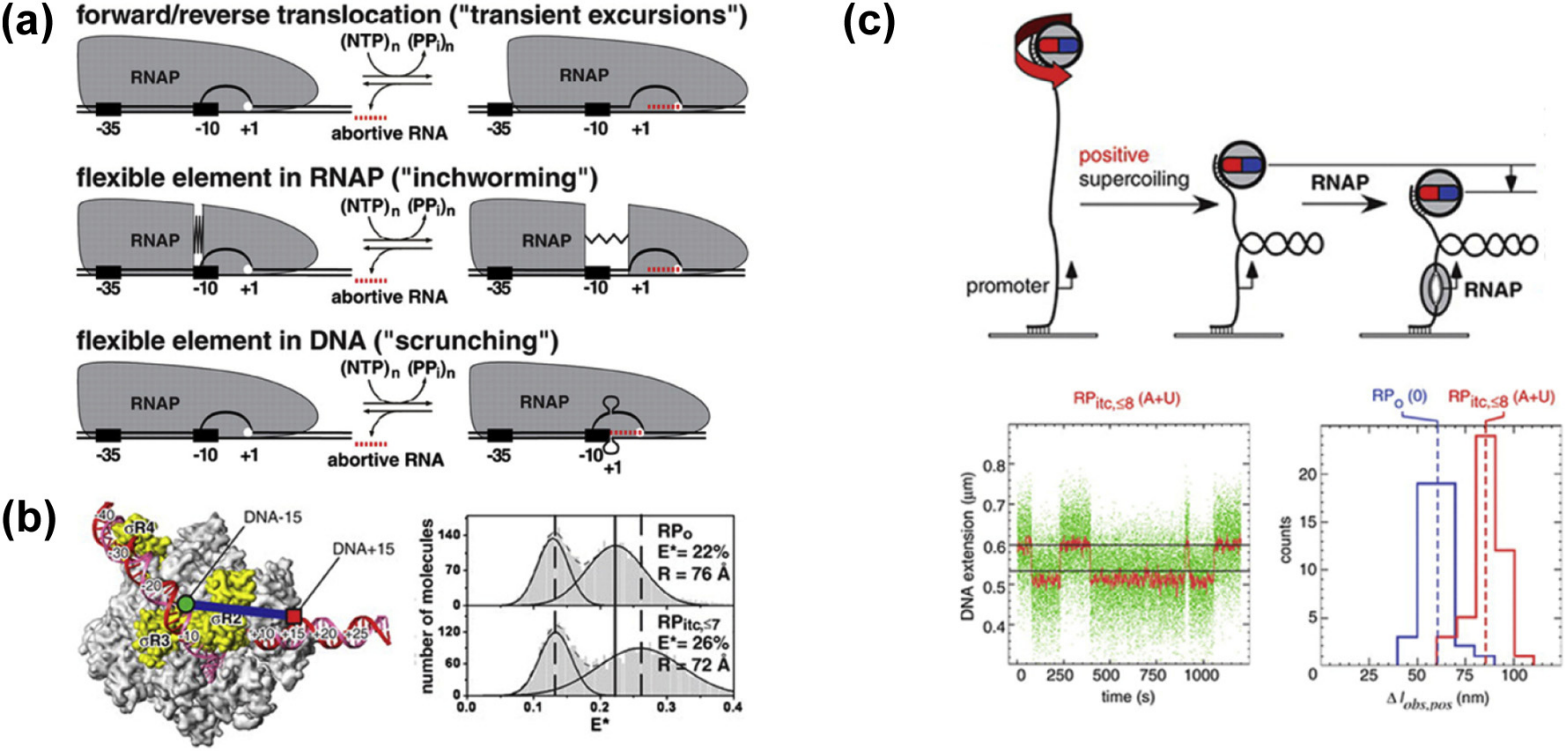
A) Schematic of the three proposed models for mechanism of initial transcription: (*top*), transient excursions; (*middle*) inchworming; (*bottom*) scrunching.; B) smFRET study showing scrunching during initial transcription; single molecule assay showing increasing FRET efficiency (decrease in distance) for dyes in positions − 15 (Cy3b) and + 15 (Alexa647) of a lacCONS promoter. Subpanels show E ∗ histograms of open complex (RP_o_) and initial transcribing complexes with up to 7-nt RNA (RP_ITC_, ≤ 7). The histograms show distributions of free DNA (lower- E ∗ species) and the RNA polymerase (RNAP)–DNA complexes. An increase in FRET efficiency indicates a decrease in distance in going from RP_o_ to RP_ITC_ and supports the scrunching model for initial transcription; (from Kapanidis et al, Science 2006 [64], used with permission). C) magnetic tweezer study showing 1 bp scrunching per nucleotide addition cycle during initial transcription. (*top*) the end-to-end extension of a mechanically stretched, negatively supercoiled or positively supercoiled single DNA molecule containing a single promoter is monitored. Unwinding of *n* turn of DNA by RNAP result in the compensatory loss of *n* negative supercoils or gain of *n* positive supercoils and a readily detectable movement of the bead; (*bottom*) magnetic tweezers data showing scrunching of DNA during initial transcription (adapted from Revyakin et al, Science, 2006 [65]; used with permission).

After formation of the stable open complex, the polymerase starts cycles of de novo RNA synthesis, which can end in either productive or abortive RNA synthesis. In the productive pathway, RNAP synthesises RNA up to a length of 9- to 11-nt, at which point RNAP escapes from the promoter and enters elongation. In the abortive pathway (also known as abortive initiation), RNAP synthesises short RNA fragments, but instead of escaping from the promoter, RNAP releases short RNAs, reverts back to RP_o_, and re-initiates RNA synthesis [60], [61]. The balance between productive and abortive pathways depends on the promoter and initial transcribed sequences [62], [63]. Investigation of the mechanism of initial transcription (including the mechanisms of abortive initiation and promoter escape) has been powered by advances in single-molecule fluorescence and DNA nano-manipulation methods. Proposed models for initial transcription included models envisioning either translocation of the polymerase (transient excursion model) or a flexible element in the polymerase (inch-worming model) or a flexible element in the DNA (scrunching model) (Fig. 3A). Initial studies using confocal smFRET methods measured distances within a RNAP-promoter complex engaged in initial transcription and showed that, during initial transcription, downstream template DNA was pulled into the active site cleft, in a process termed “DNA scrunching”, whereas RNA polymerase remained stationary on the promoter DNA fragment, resulting in an increase in the length of the transcription bubble (Fig. 3B) [64]. A parallel magnetic-tweezers study on similar RNAP-promoter complexes also showed that the transcription bubble expanded in a RNA-length-dependent manner revealing that the DNA is pulled into the active centre cleft by 1 bp per nucleotide addition cycle following formation of the initial di-nucleotide (Fig. 3C) [65]; the magnetic tweezers study also established scrunching to be an obligatory step in initial transcription for efficient promoter escape. The results of these two single-molecule studies supported the model which invoked a flexible element in the DNA (scrunching model) wherea “stressed intermediate” is formed during initial transcription, with accumulated DNA-unwinding and DNA-compaction stress, and in which accumulated stress is used to drive breakage of interactions between RNAP and promoter DNA and between RNAP and σ_70_ during promoter escape.

More recent studies using crosslinking approaches within RP_o_ has raised the possibility that for some promoters (e.g. *rrn*BP1), there may be scrunching in absence of nucleotides in the RP_o_ leading to unusual transcription start sites. The crosslinking work also proposed that scrunching in RP_o_ prepares the complex for efficient promoter escape and is a major determinant for ensuring high transcription turnovers at these promoters [66], [67]. Similar studies using a crosslinking mapping approach in initial transcribing complexes revealed that the scrunched DNA strands in RP_itc_ (one with a 5-mer RNA) shared different fates, with the non-template strand bulging out into the solvent, while the scrunched template strand remained within the polymerase creating stress on interactions with the β’ clamp and σR3.2 [68].

### σR3.2 finger and pausing in initial transcription

**Fig. 4:**
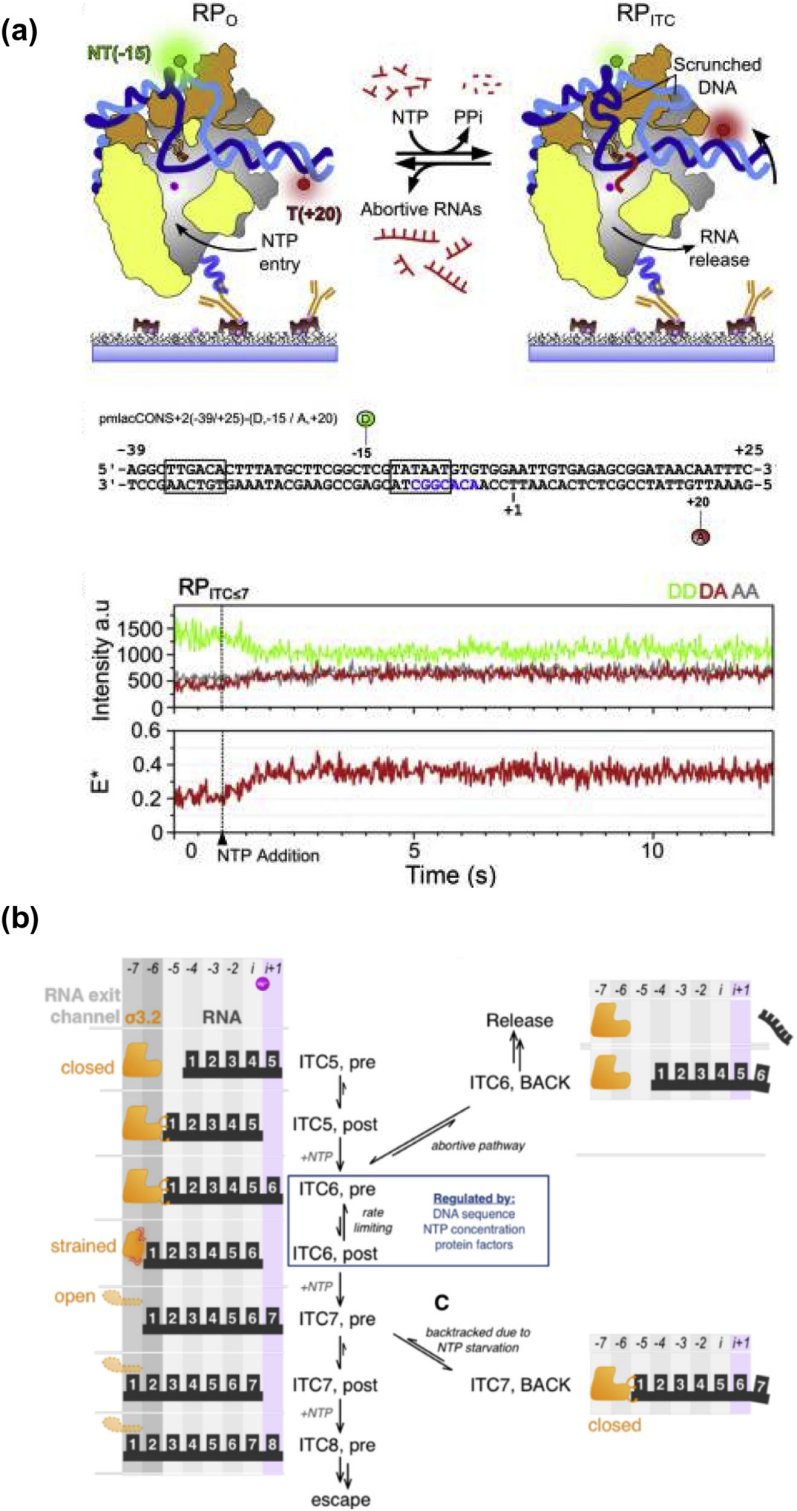
A) smFRET assay showing pausing in initial transcription; (*top*): left, RP_o_; right, initial transcribing complex (ITC). Donor is in green; acceptor in red; σ^70^ in orange; RNAP in grey, except for the β subunit (omitted for clarity) and regions protruding from the cut-away plane (in yellow); template strand in blue; non-template strand in teal; nascent RNA in red; and RNAP active site in pink. The penta-His antibody anchors RP_o_ to the surface. The initial FRET efficiency is low; upon NTP addition, scrunching moves the acceptor closer to the donor, increasing FRET efficiency; (*middle*): lacCONS DNA fragment for FRET assay; the − 10/−4 pre-melted region is in blue; (*bottom*): time trace showing an increase to E ∗ ∼ 0.37 upon adding 80 μM UTP and GTP to form RP_ITC≤7_. The NTP addition point is marked with a dashed line. Frame time: 20 ms. DD trace (green trace, top), donor emission upon donor excitation; DA trace (red trace, top), acceptor emission upon donor excitation; AA trace (grey trace, top), acceptor emission upon acceptor excitation. DD and DA are used for calculating apparent FRET efficiency E ∗; B) Model for pausing in initial transcription showing the different elements in the RNAP-promoter complex in play; (*top*): productive path for initial transcription. Coloured columns show translocational registers adopted by growing RNA (in black). Binding site for incoming NTP is in light purple; σ3.2 loop is shown in three putative conformations (in orange). The translocational equilibrium for RP_ITC6_ is controlled by several regulatory factors that modulate the lifetime of paused states arising from a pre-translocated RP_ITC6_; (*middle*): abortive path for initial transcription, branching from the pre-translocated RP_ITC6_ state of the productive path; (*bottom*): path for the formation of stable backtracked scrunched states, branching from the pre-translocated RP_ITC6_ state of initial transcription during NTP starvation that limits RNA synthesis to 7 nt in length. (adapted from Duchi et al, Mol Cell, 2016 [69]; used with permission).

The σR3.2 finger was identified early on as an RNAP determinant of abortive initiation. This segment lies along the linker connecting σR3 and σR4, and passes through to the active centre cleft making contacts with template DNA [19]. Structural studies of initial transcribing complexes clearly place the σR3.2 finger along the RNA-exit channel [41]. Once RNAP starts synthesising RNA, the nascent RNA chain grows and the 3′-end of the transcript moves towards the RNA-exit channel. High-resolution crystal structures of initial transcribing complexes revealed that a transcript of 5–6 nt in length would encounter the σR3.2 finger in its path (Fig. 4A; top); beyond this point, the nascent RNA chain must either dislodge the σR3.2 from its position, or dissociate from the complex (abortive initiation) and re-start transcription. These structure-based predictions were also supported by studies showing that deletion of a 7-residue segment at the tip of the finger (a.a. 513–519) led to decrease in the amount of short transcripts [29], and studies showing that deletion of the σR3/σR4 linker in its entirety led to no abortive transcripts [13].

Recent smFRET studies investigating scrunching during initial transcription found highly stable scrunched intermediates and extensive pausing during initial transcription after synthesis of a 6-mer RNA product at a *lac* promoter (Fig. 4A, bottom) [69]. Similar experiments with RNAP containing a deletion of σR3.2 finger residues 513–519 resulted in greatly reduced (but not entirely eliminated) pausing, thus establishing the critical role played by this structural element. In addition to the barrier presented by σR3.2, it was shown that removing a promoter sequence element at + 6 to + 7 of the lac promoter (Y_−1_G_+1_) resulted in greatly reduced pausing during initial transcription [69], [70]. The particular sequence involved is similar to a consensus elemental pause sequence identified in transcription elongation (G_−10_Y_−1_G_+1_) [71] and may be operating under similar principles where the barrier provided by the G_−10_ element at the upstream end of the transcription bubble in the elongation pause is being substituted by the σR3.2 finger in the initiation pause. The observation of sequence dependence in the initiation pause is also in good agreement with previous studies which reported that promoters with certain sequence elements in the initial transcribing sequence tend to produce more abortive transcripts. A similar study using single-molecule assays of run-off transcript production on a similar *lac* promoter fragment also reported observation of long duration pausing during initial transcription [72]. Based on these observations a “working model” for initial transcription has been proposed which starts with synthesis of 2–4 nt long RNA (these products quickly dissociate). When the RNA reaches 5 nt it is stabilised most likely in a post-translocated state as RP_ITC5_. The next incoming nucleotide can then come in and bind at the i + 1 site resulting in its rapid incorporation to form a pre-translocated RP_ITC6._ The σ3.2-template strand contacts limits scrunching upto 4 nt and 5′ end of the 6-nt RNA clashes with σ3.2, preventing translocation from the pre- to post-translocated state. At this point the complex enters an off-pathway paused state (Fig. 4B).

A follow-up smFRET study explored in detail the trajectory past the pause at + 6 on the same promoter and showed that transcribing complexes exit the pause and branch into three different pathways: productive transcription, abortive release of RNA and a slow cycling between DNA conformations with different extents of scrunching without RNA release [73]. In the productive pathway GTP binds to a transiently sampled post-translocated state of RP_ITC6_ and extends RNA to a 7-mer and eventually σ3.2 is displaced by the growing RNA chain. The growing RNA also severs the contacts of σ3.2 with the template, allowing the template to scrunch further up to promoter escape. Taken together, these recent studies suggest that a complex, dynamic sequence of events underpin abortive initiation during initial transcription.

## Promoter escape and beyond

Initial transcription cycles that are not caught upon in abortive initiation cycles are able to synthesise long RNA products (9–15 nt) and result in escape of RNAP from the promoter and entry into transcription elongation. It has been observed that there is a negative correlation between the strength of the promoter and the efficiency of the promoter escape, with stronger promoters generally resulting in relatively higher yields of abortive products and lower promoter escape efficiency [74], [75], [76]. This supports the idea that for the polymerase to escape the promoter sequence specific interactions in the open complex must be broken. As discussed earlier, it has been proposed that the process of DNA scrunching is the fundamental process that underpins the process of acquisition of energy required to break the RNAP-promoter and RNAP-σ70 interactions. However, there are two additional crucial elements in determining promoter escape efficiency:

### Conflict between growing RNA chain and σR3-σR4 linker

The structures of initial transcribing complexes reveal that the RNA transcript would emerge out of the RNA-exit channel of RNAP when it is longer than 15 nt. This corresponds to an RNA length that should result in repositioning of the σR3-σR4 linker element and of σR4 from their respective positions in RNAP, leading to proposals that, once the RNA reaches a sufficiently long length, it displaces the σR3-σR4 linker and σR4, facilitating promoter escape and dissociation of σ^70^ from RNAP.

### The Initially Transcribed Sequence (ITS)

The ITS could affect promoter escape via the strength of the transcribed DNA/DNA hybrid and the strength of the resulting RNA/DNA hybrid; both factors play a role in determining open complex stability, and may also affect promoter escape efficiency. A more recent study used parallel Next Generation Sequence (NGS) approach to study the escape kinetics at a large set of ITS variants for four different promoter sequences [77]. The results of these studies show that ITS does play a critical role in determining the escape efficiency of RNAP through a combination of position-dependent effects (mainly via pausing in initial transcription as described in the previous section) and position-independent effects (mainly resulting from the strength of the DNA/DNA and DNA/RNA hybrids involved) [77].

### σ^70^-dependent pausing in elongation

**Fig. 5:**
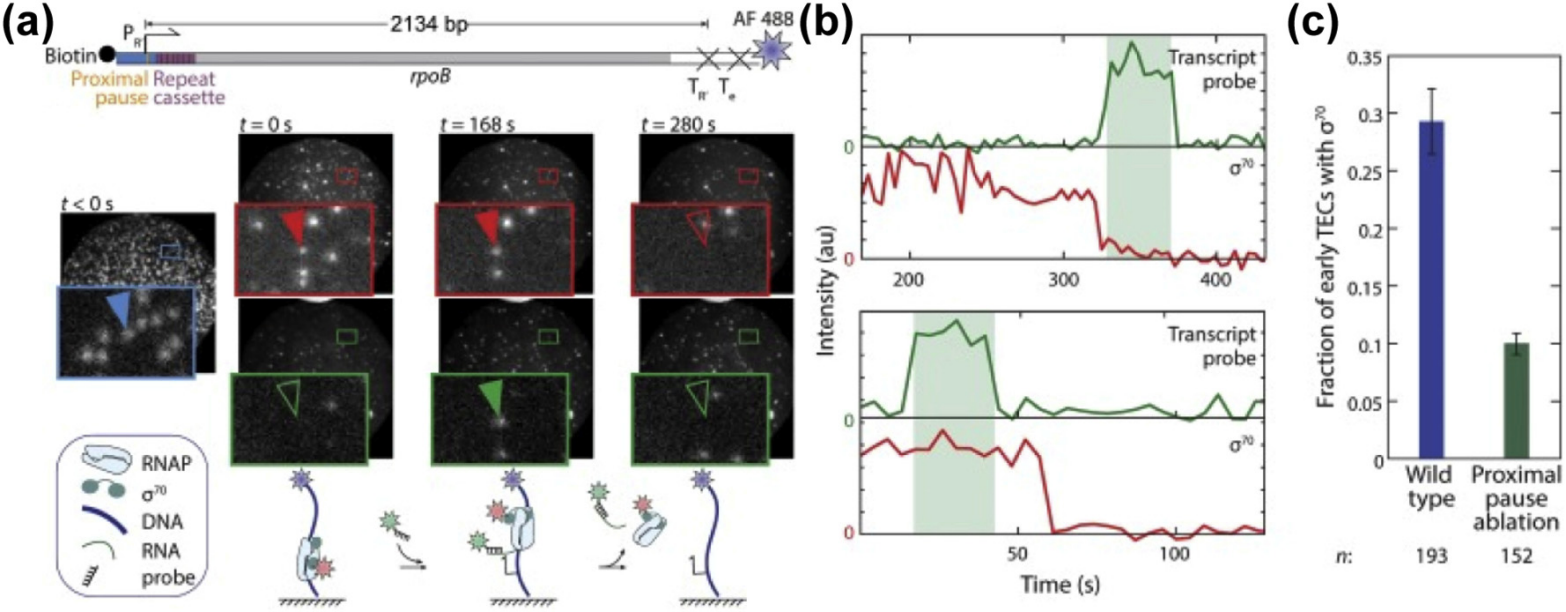
A) (*top*): Design of single molecule assay for detecting retention of σ^70^ during elongation; (*bottom*): images of the same microscope field of view of AF488–DNA (blue), Cy5–σ^70^ (red), and transcript-hybridization probe (green) taken at the specified times. B) (*top*): Two examples of time records of transcript probe (green) and σ^70^ (red) fluorescence, each colocalized at a DNA spot σ^70^ fluorescence departs before the time interval (shaded) during which transcript probe fluorescence is present. (*bottom*) σ^70^ fluorescence persists throughout transcript probe interval. (C) The fraction (± SEM) of TECs that retain σ^70^ at the time transcript probe is first detected on the TEC (reprinted from Harden et al, PNAS, 2016 [81]; used with permission).

Early studies proposed that the RNAP obligatorily lost the σ^70^ subunit following promoter escape; however, FRET-based studies on early elongation complexes found that σ^70^ could be highly retained in the elongating polymerase [78]. Subsequent smFRET experiments on diffusing and surface-immobilised transcription complexes confirmed these results, provided a quantitative assessment of σ^70^ retention in elongation complexes, and estimated the half-life of σ70 retention to be ∼ 50s [79]; this timescale is long enough for the RNAP to be able to transcribe ∼ 1 kb DNA. The findings of this study therefore indicate that for transcription of some genes at least σ70 may be retained for the entire phase of elongation. It has since then been found that RNAP molecules which retained σ^70^ in elongating complexes may recognise some − 10 or − 35-like DNA sequence element in the transcribing gene, resulting in σ^70^-dependent pausing for the elongating polymerase [80]. A recent single molecule study looking at transcription of a long gene (∼ 2000 nt) confirmed that σ70 can be retained by a substantial fraction of elongating RNAP molecules for the entire length of this very long sequence (Fig. 5). The study also found that polymerases retaining σ70 during elongation exhibit pausing due to recognition of promoter like elements hundreds of nucleotides downstream of the promoter [81].
